# Supplementary material for: Adaptation and validation of simple tools to screen and monitor for oral PrEP adherence
Source: PLoS One. 2021 May 27;16(5):e0251823. doi: 10.1371/journal.pone.0251823 (PMC8158999; doi:10.1371/journal.pone.0251823)
Supplement: Attachment — Submitted filename: PONE-D-21-03228_reviewer (1).pdf [file pone.0251823.s001.pdf]

PLOS ONE

Adaptation and validation of simple tools to screen and monitor for oral PrEP adherence

--Manuscript Draft--

**Manuscript Number:**	PONE-D-21-03228
**Article Type:**	Research Article
**Full Title:**	Adaptation and validation of simple tools to screen and monitor for oral PrEP adherence
**Short Title:**	Screening and adherence monitoring tools for oral PrEP
**Corresponding Author:**	Elizabeth E. Tolley, PhD, MA<br>FHI 360<br>Durham, NC UNITED STATES
**Keywords:**	Adherence; HIV prevention; Clinical trial; Screening Tool; Monitoring Tool; Adolescents; Women; sub-Saharan Africa; Psychometric Assessment
**Abstract:**	Introduction<br><br>Oral, vaginal and other pre-exposure prophylaxis (PrEP) products for HIV prevention are in various stages of development. Low adherence poses a serious challenge to successful evaluation in trials. In a previous study, we developed tools to screen for general adherence and specifically monitor intravaginal ring adherence within the context of HIV prevention clinical trials. This study aimed to further validate the screening tool and to adapt and provide initial psychometric validation for an oral pill monitoring tool.<br><br>Materials and methods<br><br>We administered a cross-sectional survey between June and October 2018 at a trial site located near Cape Town, South Africa, and another in Thika, Kenya, with 193 women who had experience using daily oral pills. We fit confirmatory factor analysis models on the screening tool items to assess our previously-hypothesized subscale structure. We conducted an exploratory factor analysis of oral PrEP monitoring items to determine the underlying subscale structure. We then assessed the construct validity of each tool by comparing subscales against each other within the current sample and against our original sample.<br><br>Results<br><br>The screening tool structure showed moderate evidence of construct validity. As a whole, the tool performed in a similar way to the original sample. The monitoring tool items, which were revised to assess perceptions about and experiences using daily oral PrEP, factored into five subscales that showed moderate to good reliability. Four of the five subscales had a similar structure overall to the vaginal ring monitoring tool from which they were adapted.<br><br>Conclusions<br><br>Accurate measurement of HIV-prevention product adherence is of critical importance to the assessment of product efficacy and safety in clinical trials, and the support of safe and effective product use in non-trial settings. In this study, we provide further validation for these measures, demonstrating the screening tool’s utility in additional populations and adapting the monitoring tool’s utility for different HIV-prevention products.
**Order of Authors:**	Elizabeth E. Tolley, PhD, MA
	Seth Zissette
	Andres Martinez

Powered by Editorial Manager® and ProduXion Manager® from Aries Systems Corporation

	Homaira Hanif
	Katherine Gill
	Nelly Mugo
	Laura Myers
	Ednar Casmir
	Menna Duyver
	Kenneth Ngure
	Gustavo F. Doncel
**Additional Information:**	
**Question**	**Response**
**Financial Disclosure**<br><br>Enter a financial disclosure statement that describes the sources of funding for the work included in this submission. Review the submission guidelines for detailed requirements. View published research articles from *PLOS ONE* for specific examples.<br><br>This statement is required for submission and **will appear in the published article** if the submission is accepted. Please make sure it is accurate.<br><br>**Unfunded studies**<br>Enter: *The author(s) received no specific funding for this work.*<br><br>**Funded studies**<br>Enter a statement with the following details:<br>• Initials of the authors who received each award<br>• Grant numbers awarded to each author<br>• The full name of each funder<br>• URL of each funder website<br>• Did the sponsors or funders play any role in the study design, data collection and analysis, decision to publish, or preparation of the manuscript?<br>• **NO** - Include this sentence at the end of your statement: *The funders had no role in study design, data collection and analysis, decision to publish, or preparation of the manuscript.*<br>• **YES** - Specify the role(s) played.<br><br>* typeset	This study was funded by the U.S. President's Emergency Plan for AIDS Relief (PEPFAR) and the U.S. Agency for International Development (USAID) through a cooperative agreement (AID-OAA-A-14-00011) with CONRAD and Eastern Virginia Medical School. The funders had no role in study design, data collection and analysis, decision to publish, or preparation of the manuscript.
**Competing Interests**	The authors have declared that no competing interests exist.

Use the instructions below to enter a competing interest statement for this submission. On behalf of all authors, disclose any competing interests that could be perceived to bias this work—acknowledging all financial support and any other relevant financial or non-financial competing interests.<br><br>This statement is required for submission and **will appear in the published article** if the submission is accepted. Please make sure it is accurate and that any funding sources listed in your Funding Information later in the submission form are also declared in your Financial Disclosure statement.<br><br>View published research articles from *PLOS ONE* for specific examples.<br><br>**NO authors have competing interests**<br><br>Enter: *The authors have declared that no competing interests exist.*<br><br>**Authors with competing interests**<br><br>Enter competing interest details beginning with this statement:<br><br>*I have read the journal's policy and the authors of this manuscript have the following competing interests: [insert competing interests here]*<br><br>* typeset	
**Ethics Statement**<br><br>Enter an ethics statement for this submission. This statement is required if the study involved:<br><br>• Human participants<br>• Human specimens or tissue<br>• Vertebrate animals or cephalopods<br>• Vertebrate embryos or tissues	Ethical approval for this project was obtained from the FHI 360 Protection of Human Subjects Committee (protocol 1151358), the University of Cape Town Health Research Ethics Committee, and the Kenya Medical Research Institute Scientific and Ethics Review Unit. Written consent was obtained electronically from each participant before each survey. Data were de-identified to protect participant confidentiality.

• Field research<br><br>Write "N/A" if the submission does not require an ethics statement.<br><br>General guidance is provided below. Consult the submission guidelines for detailed instructions. **Make sure that all information entered here is included in the Methods section of the manuscript.**	

**Format for specific study types**<br><br>**Human Subject Research (involving human participants and/or tissue)**<br>• Give the name of the institutional review board or ethics committee that approved the study<br>• Include the approval number and/or a statement indicating approval of this research<br>• Indicate the form of consent obtained (written/oral) or the reason that consent was not obtained (e.g. the data were analyzed anonymously)<br><br>**Animal Research (involving vertebrate animals, embryos or tissues)**<br>• Provide the name of the Institutional Animal Care and Use Committee (IACUC) or other relevant ethics board that reviewed the study protocol, and indicate whether they approved this research or granted a formal waiver of ethical approval<br>• Include an approval number if one was obtained<br>• If the study involved *non-human primates*, add *additional details* about animal welfare and steps taken to ameliorate suffering<br>• If anesthesia, euthanasia, or any kind of animal sacrifice is part of the study, include briefly which substances and/or methods were applied<br><br>**Field Research**<br><br>Include the following details if this study involves the collection of plant, animal, or other materials from a natural setting:<br>• Field permit number<br>• Name of the institution or relevant body that granted permission	
**Data Availability**<br><br>Authors are required to make all data underlying the findings described fully available, without restriction, and from the time of publication. PLOS allows rare exceptions to address legal and ethical concerns. See the PLOS Data Policy and FAQ for detailed information.	Yes - all data are fully available without restriction

A Data Availability Statement describing where the data can be found is required at submission. Your answers to this question constitute the Data Availability Statement and **will be published in the article**, if accepted.<br><br>**Important:** Stating 'data available on request from the author' is not sufficient. If your data are only available upon request, select 'No' for the first question and explain your exceptional situation in the text box.<br><br>Do the authors confirm that all data underlying the findings described in their manuscript are fully available without restriction?	
**Describe where the data may be found in full sentences. If you are copying our sample text, replace any instances of XXX with the appropriate details.**<br><br>• If the data are **held or will be held in a public repository**, include URLs, accession numbers or DOIs. If this information will only be available after acceptance, indicate this by ticking the box below. For example: *All XXX files are available from the XXX database (accession number(s) XXX, XXX.)*.<br>• If the data are all contained **within the manuscript and/or Supporting Information files**, enter the following: *All relevant data are within the manuscript and its Supporting Information files.*<br>• If neither of these applies but you are able to provide **details of access elsewhere**, with or without limitations, please do so. For example:<br><br>*Data cannot be shared publicly because of [XXX]. Data are available from the XXX Institutional Data Access / Ethics Committee (contact via XXX) for researchers who meet the criteria for access to confidential data.*<br><br>*The data underlying the results presented in the study are available from (include the name of the third party*	Our data will be held on the Harvard DataVerse repository. We will provide the url once the paper is under review.

and contact information or URL).<br>• This text is appropriate if the data are owned by a third party and authors do not have permission to share the data.<br><br>* typeset	
Additional data availability information:	Tick here if the URLs/accession numbers/DOIs will be available only after acceptance of the manuscript for publication so that we can ensure their inclusion before publication.

Click here to access/download;Manuscript;Adherence tools to oral PrEP_Final_Jan292021.docx

Full Title: Adaptation and validation of simple tools to screen and monitor for oral PrEP [2]adherence

Short Title: Screening and adherence monitoring tools for oral PrEP

Seth Zissette[1,5]; Elizabeth E. Tolley[1*]; Andres Martinez[1]; Homaira Hanif[2]; Katherine Gill[3]; Nelly Mugo[4]; Laura Myers[3]; Ednar Casmir[4]; Menna Duyver[3]; Kenneth Ngure[6], Gustavo F. Doncel[2]

[1]FHI 360, Behavioral, Epidemiological, and Clinical Sciences, Durham, United States
[2]CONRAD, Norfolk, United States
[3]Desmond Tutu HIV Foundation, University of Cape Town, Cape Town, South Africa
[4]Kenya Medical Research Institute, Nairobi, Kenya
[5]University of Notre Dame, South Bend, United States
[6]Jomo Kenyatta University of Agriculture and Technology, Nairobi, Kenya

*Corresponding author
E-mail: btolley@fhi360.org (ET)

DRAFT

Abstract

Introduction

Oral, vaginal and other pre-exposure prophylaxis (PrEP) products for HIV prevention are in various stages of development. Low adherence poses a serious challenge to successful evaluation in trials. In a previous study, we developed tools to screen for general adherence and specifically monitor intravaginal ring adherence within the context of HIV prevention clinical trials. This study aimed to further validate the screening tool and to adapt and provide initial psychometric validation for an oral pill monitoring tool.

Materials and methods

We administered a cross-sectional survey between June and October 2018 at a trial site located near Cape Town, South Africa, and another in Thika, Kenya, with 193 women who had experience using daily oral pills. We fit confirmatory factor analysis models on the screening tool items to assess our previously-hypothesized subscale structure. We conducted an exploratory factor analysis of oral PrEP monitoring items to determine the underlying subscale structure. We then assessed the construct validity of each tool by comparing subscales against each other within the current sample and against our original sample.

Results

The screening tool structure showed moderate evidence of construct validity. As a whole, the tool performed in a similar way to the original sample. The monitoring tool items, which were revised to assess perceptions about and experiences using daily oral PrEP, factored into five subscales that showed moderate to good reliability. Four of the five subscales had a similar structure overall to the vaginal ring monitoring tool from which they were adapted.

Conclusions

Accurate measurement of HIV-prevention product adherence is of critical importance to the assessment of product efficacy and safety in clinical trials, and the support of safe and effective product use in non-trial settings. In this study, we provide further validation for these measures, demonstrating the screening tool's utility in additional populations and adapting the monitoring tool's utility for different HIV-prevention products.

Introduction

New biomedical methods for HIV prevention have changed the landscape of the epidemic over the past decade. Daily oral regimens of pre-exposure prophylaxis (PrEP) have proven effective in reducing transmission [1-3] and been introduced in multiple settings [4, 5]. Additional HIV-only and multipurpose prevention products, such as vaginal rings, long-acting injectables, implants, and HIV vaccines are at various stages in the development pipeline as well; as of October 2019, the AIDS Vaccine Advocacy Coalition identified over 50 ongoing or planned HIV prevention product trials [6, 7]. Over one third of these are focused primarily or significantly on use by women, who often face disproportionate burdens of the disease and unique challenges to utilizing prevention products [6, 8].

As PrEP and other HIV prevention products become more widely available, product adherence poses a serious challenge to their successful use, particularly among young African women [6, 8, 9]. In trials of PrEP effectiveness, sub-analyses indicated that poor adherence to the prescribed dosing regimen reduced effectiveness [10, 11]. The importance of adherence to product use

DRAFT

instructions was underscored by results from the VOICE and FEM-PrEP trials, where poor adherence to vaginal (VOICE) and oral (VOICE and FEM-PrEP) dosing regimens undermined the trials' ability to determine effectiveness [12, 13]. Validated tools that enable trial implementers to proactively screen for and prospectively monitor adherence-related determinants are vitally important for current and future studies.

Conventional self-reported adherence instruments within HIV prevention trials often rely on single-item questions. While inexpensive and easy to administer, they are prone to self-presentation and/or recall biases or require participants to make complex mental calculations [14, 15]. Consequently, HIV prevention trials generally prefer to use objective markers (e.g., detection of drug presence or level in blood or pill count measures) to assess product adherence [16, 17]. However, these measures also have limitations [18]. For example, biomarker measures are often expensive, may be prone to a "white coat effect" and generally measure adherence to active, but not placebo products [19]. Although studies have found some evidence of dose-response between adherence and residual drug levels in returned vaginal rings, individual variability challenges this as an approach to monitoring adherence [20]. Furthermore, biomarker measures of adherence for event-driven PrEP are not included in the literature [21, 22]. Pill counts may over or under-estimate adherence due to pill-dumping or use of pill cases [23]. Scales are composed of multiple items in the form of questions or statements that, when combined, measure a more complex construct that may not be directly observable [24]. When psychometrically validated, such measures may better assess the multiple factors contributing to adherence/non-adherence [25-28].

DRAFT

In a previous study conducted in four South African sites [9], two different tools were developed to screen for and monitor product-related adherence within the context of clinical trial participation. The screening tool comprises six subscales measuring potential participants' attitudes about, and social and structural challenges towards trial participation. Items were intended to be administered prior to product use and were product-agnostic. The monitoring tool comprised four sets of items intended to measure specific attitudes related to using product over trial participation. In the previous study, three versions of the monitoring items were developed relating to use of a vaginal ring, vaginal gel and oral pills. Although the vaginal ring items were validated, the survey sample was insufficiently powered to validate the vaginal gel or oral pill monitoring items. Previous research has shown the degree to which product type can influence product acceptability and adherence [29-31], necessitating the need for product-specific adaptations for adherence monitoring. In order to facilitate the use of the screening and monitoring tools in upcoming trials of novel antiretroviral (ARV) oral pills, we sought to refine and validate both the screening tool and the oral pill monitoring tool items.

The overall goal of this project was to adapt and provide further psychometric validation for the screening tool and initial psychometric validation for the oral pill monitoring tool. Starting with the tools developed in the aforementioned study, we used scale development processes to adapt the screening and monitoring tool items for local context in two sites in South Africa and Kenya, including rounds of cognitive interviewing to improve item framing, comprehensibility and salience, followed by administration of a survey to facilitate both confirmatory and exploratory factor analyses and psychometric evaluation of resulting constructs.

DRAFT

Materials and methods

Data collection

We administered a cross-sectional survey between June and October 2018 in two trial sites located near Cape Town, South Africa, and Thika, Kenya. The study protocol was approved by FHI360's and local IRBs in both countries. The survey was administered face-to-face by data collectors who electronically captured responses on tablets with a total of 193 women. Participants were eligible for recruitment if they were female, between the ages of 18-30, had experience using daily oral pills (any indication), and provided written consent electronically prior to the survey. Because potential trial participants for whom our scales are intended might comprise those with and without previous experience in clinical research, we recruited both former trial participants (FTP) from previous or ongoing trials of oral PrEP and trial-naïve participants (TNP). Participants could elect to hear and respond to any part of or all of the survey in either English or their local language (isiXhosa or Kiswahi[illegible]

The survey contained three primary sections. Following basic demographic and past trial experience questions to determine eligibility, the first section included 54 items in random order making up the six subscales of the screening tool and was administered uniformly to all women. The second section comprised of 45 monitoring items in random order adapted for oral PrEP. There were two versions of this section; one focused on oral PrEP and administered only to women who had or were participating in oral PrEP trials, the other version adapted oral PrEP-specific items to other types of oral pill use to better fit the experiences of trial-naïve women. All items in the screening tool and monitoring tool sections were asked on a 6-point Likert scale with responses ranging from "1=Disagree a lot" to "6=Agree a lot". The third section included

DRAFT

additional demographic and psychosocial variables, such as age, education level, income status, and relationship status and dynamics.

Data Analysis

Screening Tool

Our original tool comprised a total of 59 items organized into six domains, which consisted of factors that measured reasons for clinical trial participation (Commitment to Research, Personal & Health Benefits), beliefs about the trial and product (Distrust of Research) and agency (Trial Incompatibility, Partner Disclosure, Visit Adherence). Because the tool was intended to be administered prior to a trial participant's randomization to product, the items are product agnostic. Therefore, we aimed to further validate the tool by determining how similarly the items performed in our new study samples relative to our original study.

Following cognitive interviews with 36 women to contextually adapt the items, we retained 54 items relevant in both settings for use in the survey. Using only these items from the survey data, we started by fitting a confirmatory factor analysis (CFA) model reflecting the hypothesized 6-factor structure to the full new sample of participants. We then repeated the analysis using only the sample of former-trial participants. Rather than treat items as continuous as is common in standard CFAs, we treated the response scale of all the items as ordinal to reflect its Likert nature.

Monitoring Tool

DRAFT

Our original monitoring tool comprised of 48 vaginal ring-specific items organized into 4 domains, relating to vaginal ring doubts, benefits, side effects and removal. We assumed that the new and/or revised items relating to oral pill adherence might not fit the same thematic structure as the earlier vaginal ring items.

For this analysis, we used only the 45 monitoring items included in the survey. We examined the response distribution, means, standard deviations, skew, and kurtosis of all items, with the aim of eliminating items that exhibited high skew or little variation as evidenced by flooring/ceiling effects, low standard deviation, or high skew/kurtosis. We then conducted exploratory factor analyses (EFA) of monitoring items. We ran an EFA with oblique (Promax) rotation and an unrestricted number of factors to determine the possible number of factors. We then examined the scree plot and table of eigenvalues to identify the number of factors with eigenvalues greater than 1, as well as observing the least diminishing difference between next eigenvalue, to assess how many factors should be extracted.

After determining a possible acceptable range for number of factors, we generated each possible solution iteratively. This involved the following steps:

- Conducting EFAs with oblique (Promax) rotation and dropping items with loadings less than 0.4 for each possible solution with the specified number of factors.
- Dropping any items that did not load to any factor in any solution and re-running EFAs until all included items loaded onto at least one factor in at least one solution.
- Assessing reliability using Cronbach's alpha with a preferred threshold of 0.7 and content validity of each factor in each solution.

DRAFT

- Comparing factor solutions with previous vaginal ring monitoring subscales to determine the best possible solution.

Construct Validity

We assessed tool validity using a multifaceted approach. We first compared the final proposed screening tool and monitoring tool against their original versions, focusing on reliability and number of items. We then compared Pearson Product Moment correlations between the screening tool and monitoring tool within and between the original and current samples to determine whether the scales were performing in a predictable manner. We considered differences of 0.20 and higher to indicate relationships between subscales that differed from the original sample. Finally, we examined descriptive statistics for subscale scores by subsamples (site and trial participation status), generating scores by averaging item responses within each subscale.

Ethical Considerations

Ethical approval for this project was obtained from the FHI 360 Protection of Human Subjects Committee (protocol 1151358), the University of Cape Town Health Research Ethics Committee, and the Kenya Medical Research Institute Scientific and Ethics Review Unit. Written consent was obtained electronically from each participant before each survey. Data were de-identified to protect participant confidentiality.

Results

DRAFT

Sample characteristics

In general, the women who participated in our survey were young, with a mean age of 22 (Table 1). Women in South Africa were on average two years younger than women in Kenya and the majority (66%) lived with parents and other family members, whereas just over a third of Kenyan participants (35%) lived with parents. Just over one-quarter of South African women compared to almost one-half of Kenyans had at least one child. In both sites, former or current trial participants tended to be younger than women who had not participated in trials before. Most participants in both samples were sexually active with a regular partner, although they were not necessarily living with the partner. Women were eligible to participate in this study if they were currently or had been on a daily pill regimen, including daily contraceptive pills, daily vitamins or medications for another conditions, as well as PrEP. We included a wide range of products with the goal of validating our "oral daily pills" adherence items while also recognizing that some women who choose to join future prevention trials and would therefore respond to our scale items will never have used daily PrEP. Nevertheless, almost 80% had participated in trials previously and more than 80% of participants had used PrEP at some point in their past – either within a trial or as a prevention option outside of a trial.

DRAFT

Table 1. Key socio-demographic information for survey sample.

	South Africa			Kenya			Total		
	TNP (n=8)	FTP (n=85)	All (n=93)	TNP (n=32)	FTP (n=68)	All (n=100)	TNP (n=40)	FTP (n=153)	Sample (n=193)
Age (years, mean)	21.5	20.8	20.9	25.0	22.1	23.1	24.3	21.4	22.0
Education (%)									
Primary or less	0	7.1	6.5	18.2	18.0	18.1	13.3	11.2	11.5
Some secondary	0	30.6	28.0	22.7	12.0	15.3	16.7	23.7	22.4
Secondary complete	50.0	29.4	31.2	9.1	8.0	8.3	20.0	21.5	21.2
College or higher	50.0	33.0	34.4	50.0	62.0	58.3	50.0	43.7	44.9
Relationship status (%)									
(Living as/) Married	25.0	7.1	8.6	45.5	6.0	18.1	40.0	6.7	12.7
Regular partner (not living together)	50.0	68.2	66.7	50.0	58.0	55.6	50.0	64.4	61.8
Sexually active, no regular partner(s)	12.5	11.8	11.8	0	30.0	20.8	33.3	18.5	15.8
Not sexually active	12.5	12.9	12.9	4.6	6.0	5.6	6.7	10.4	9.7
Currently living with (%)									
Parent/s	75.0	64.7	65.6	36.4	34.0	34.7	46.7	53.3	52.1
Other family	0	22.4	20.4	9.1	12.0	11.1	6.7	18.5	16.4
Partner	12.5	2.4	3.2	40.9	12.0	20.8	33.3	5.9	10.9
Children	0	4.7	4.3	36.4	22.0	26.4	26.7	11.1	13.9
Other	12.5	7.1	7.5	13.6	36.0	29.2	13.3	17.8	17.0
Have children (%)	50.0	25.9	28.0	68.2	38.0	47.2	63.3	30.4	36.4
Type of pill used+ (%)									
Oral contraceptive	62.5	16.5	20.4	40.6	0	13.0	45.0	9.2	16.6
Vitamin	12.5	1.2	2.2	6.3	0	2.0	7.5	0.7	2.1
Chronic health medication	0	3.5	3.2	12.5	0	4.0	10.0	2.0	3.6
PrEP	25.0	78.8	74.2	40.6	100	81.0	37.5	88.2	77.7
Used oral pills to prevent HIV (%)	25.0	87.1	81.7	46.9	100	83.0	42.5	92.8	82.4

DRAFT

Past trial participation (%)			91.4			68.0			79.3
Circumstances for PrEP use+ (%)									
Prescribed PEP	0	2.4	2.2	0	0	0*	0	1.3	1.0
PrEP trial (past)	37.5	42.4	41.9	0	0	0*	7.5	23.5	20.2
PrEP trial (current)	25.0	48.2	46.2	0	100	68.0*	5.0	71.2	57.5
Other	37.5	7.1	9.7	100	0	32.0*	87.5	3.9	21.2

+More than one response possible

DRAFT

Screening Tool

The root mean square error of approximation (RMSEA) for CFA model was 0.09, suggesting the 6-factor model was supported by the data using a 0.10 cut-off score. However, not all the goodness of fit statistics showed an acceptable fit (Table 2). Particularly, the Comparative Fit Index (CFI) – which should be at 0.90 or higher - was at inadequate levels (0.80 for the model on the full sample and 0.78 for the model on the FTP subset). To explore this further, we fit a separate CFA model to each one of the first five factors, studied the modification indices of the overall CFA fit, and re-did the analysis treating the item responses as continuous.

Table 2. Screening Tool CFA model fit statistics.

Sample	Factor	N	N Items	Chi Sq.	RMSEA [95% CI]	CFI
All	All 6 factors	179	40	1746.9***	0.09 [0.08, 0.09]	0.80
All	1. Commitment to Research	186	8	39.2**	0.07 [0.04, 0.11]	0.92
All	2. Personal & Health Benefits	189	5	45.9***	0.21 [0.16, 0.27]	0.87
All	3. Distrust of Research	192	11	44.5	0.01 [0, 0.05]	1.00
All	4. Trial Incompatibility	186	8	20.4	0.01 [0, 0.06]	1.00
All	5. Partner Disclosure	191	5	3.33	0 [0, 0.08]	1.00
All	6. Visit Adherence		3	NA	NA	NA
FTP	All 6 factors	140	38	1699.7***	0.10 [0.09, 0.11]	0.78
FTP	1. Commitment to Research	146	8	37.5***	0.08 [0.04, 0.12]	0.91
FTP	2. Personal & Health Benefits	150	7	61.4***	0.15 [0.11, 0.19]	0.88
FTP	3. Distrust of Research	152	10	36.8	0.02 [0.00, 0.06]	1.00
FTP	4. Trial Incompatibility	148	8	41.2***	0.08 [0.05, 0.12]	0.80
FTP	5. Partner Disclosure	151	5	1.49	0.00 [0.00, 0.04]	1.00
FTP	6. Visit Adherence		3	NA	NA	NA

*p<0.10, **p<0.05, ***p<0.01
Note: Results for factor Visit Adherence (Factor 6) not included as it only has 3 items.
Note: Results for the TNP sample not included due to small sample size (n=40).

DRAFT

The factors Distrust of Research, Trial Incompatibility and Partner Disclosure were the best-fitting factors. When using the full sample, the goodness-of-fit statistics for the models on these factors were all at commonly-accepted levels (RMSEA upper bound below 0.10; CFI above 0.90). The factor Commitment to Research did not fit the data as well as other factors but was still at an acceptable level (RMSEA upper bound is high at 0.11; CFI above 0.90). For the Personal and Health Benefits factor, the CFI was low and the RMSEA was high, suggesting the model did not fit the data in this case.

Monitoring Tool

Items performed well overall, with only one item being dropped due to a ceiling effect. The remaining 44 items were included in EFAs. The scree plot showed six factors with eigenvalues of 2 or more before a relatively large drop to the seventh factor. In addition, the eigenvalue analysis indicated possible 3-, 4-, 5-, or 6-factor solutions.

Multiple possible solutions were assessed. Below we present the final set of factors and items (Table 3). We chose to use three factors from the 6-factor solution, as well as two factors from the 5-factor solution, because they aligned well with our previous vaginal ring monitoring tool and had acceptable psychometric properties.

Table 3. Final (selected) solution derived from the 5- and 6-factor solutions.

Item	Loading
**Factor 1 - Study Pill Challenges (Cronbach's α=0.80)**	
1. I am not always sure that I take the study pill correctly.	0.67
2. Sometimes I feel that research staff think I don’t understand how to use the study pill.	0.62

DRAFT

3. I think there are times when the research staff is disappointed in me.	0.60
4. Using the pill every day is too difficult.	0.54
5. I sometimes have trouble taking the study pill because it is too large to swallow.	0.52
6. Sometimes, I take the study pill earlier or later than normally.	0.51
7. I sometimes forget to take the study pill for several days at a time.	0.49
8. Sometimes, I only take the study pill a few days before coming for a clinic visit.	0.45
9. I believe that there is a chance that the study pill might cause me harm.	0.44
10. I sometimes forget to attend my research visits.	0.44
11. Getting to the clinic appointments is a challenge.	0.42
12. I have doubts that using the study pill will help me.	0.41
**Factor 2 - Concerns about Side Effects (Cronbach's α=0.78)**	
1. Side effects make me want to stop using the study pill.	0.81
2. I think about stopping use of the study pill when side effects begin to interfere with daily activities.	0.72
3. The pill side effects interfere with my sex life.	0.55
4. Study pill side effects interfere with my everyday life.	0.53
5. Sometimes, if I feel worse when I take the study pill, I stop using it for a while.	0.49
6. The study pill interferes with my sleep at night.	0.44
7. I do not take the study pill when I do not feel well.	0.43
**Factor 3* - Positive Pill Adherence (Cronbach's α=0.71)**	
1. I am very confident that I can participate in research visits, even if I experience difficulties.	0.70
2. I am able to deal with any problems in following my study requirements.	0.60
3. I am very confident that I can attend clinic visits even if the visits interfere with my daily activities.	0.59
4. Using the study pill to prevent HIV/pregnancy/illness is better than using condoms.	0.53
5. The study pill makes my sex life better.	0.51
6. There are more benefits of using the study pill than difficulties.	0.51
7. Using the study pill makes me feel good about myself.	0.50
8. I am sure I can discuss honestly with the clinic staff any problems related to the study pill.	0.48
9. I am completely sure that I can continue to use the study pill on days when I do not feel well.	0.43
**Factor 4 - Social Difficulties with Clinical Trial Participation (Cronbach's α=0.73)**	
1. It is difficult for me to explain the research to my family.	0.82

DRAFT

2. It is difficult for me to explain the research to my friends.	0.77
3. I do not want to be seen with the study pill container.	0.63
**Factor 5* - Pill Benefits (Cronbach's α=0.68)**	
1. I believe that the study pill will reduce my chance of getting HIV/pregnant/ill.	0.74
2. I believe that my risk for HIV/pregnancy/illness is less when I am using the study pill.	0.73
3. I believe that I might get HIV/pregnant/ill if I don't use the pill as instructed.	0.47
4. I believe that my risk for HIV/pregnancy/illness is the same whether I used the study pill or not. [*Reverse scored*]	-0.64

*From 5-factor solution

Construct Validity

Tables 4 and 5 below provide comparisons between the original screening and monitoring tools and the final proposed subscales from the current sample. We would expect for the reliability statistic on a scale to vary across samples. In general, these statistics are similar across the original and our current samples for both the screening tool and the monitoring tool, except in the case of the screening tool's Trial Incompatibility subscale and, to a lesser extent, the Partner Disclosure subscale.

DRAFT

Table 4. Comparison of Screening Tool subscales between sample of original creation and current sample.

Subscale	Number of Items		Reliability (Cronbach's α)		Item Example
	Original	Current	Original	Current	
Commitment to Research	8	8	0.61	0.59	"The idea of participating in research is appealing to me."
Personal & Health Benefits	5	5	0.59	0.56	"I want to participate in HIV prevention research because I want free health care."
Distrust of Research	11	11	0.78	0.75	"I do not trust research in general."
Trial Incompatibility	8	8	0.69	0.51	"People who participate in HIV prevention research may be rejected by others."
Partner Disclosure	5	5	0.80	0.68	"My partner knows that I am participating in the research."
Visit Adherence	3	3	0.67	0.62	"I have never been late for an appointment."

Table 5. Comparison of Monitoring Tool subscales between sample of original creation and current sample.

Subscale		Number of Items		Reliability (Cronbach's α)		Item Example	
Original	Current	Original	Current	Original	Current	Original	Current
Vaginal Ring Doubts	Study Pill Challenges	8	12	0.78	0.80	"It is hard to believe that using the vaginal ring will help me."	"I believe that there is a chance that the study pill might cause me harm."
Concerns about Side Effects	Concerns about Side Effects	7	5	0.61	0.78	"Side effects make it difficult for me to keep using the vaginal ring."	"Side effects make me want to stop using the study pill."

DRAFT

Vaginal Ring Removal	Positive Pill Adherence	9	8	0.82	0.71	"Sometimes, if I felt worse when I had the vaginal ring in my body, I stopped using it."	"I am completely sure that I can continue to use the study pill on days when I do not feel well."
N/A	Social Difficulties with Clinical Trial Participation	N/A	3	N/A	0.73	N/A	"It is difficult for me to explain the research to my family."
Vaginal Ring Benefits	Pill Benefits	4	9	0.75	0.68	"I believe that the vaginal ring will reduce my chance of getting HIV."	"I believe that the study pill will reduce my chance of getting HIV."

DRAFT

In general, the correlations between screening and monitoring subscales in our new sample were similar in magnitude and direction as in our original sample (Table 6). Several exceptions are highlighted in Table 6.

DRAFT

Table 6. Comparisons of current Pearson Product Moment correlations between screening tool and monitoring tool subscales with correlations from original sample.

	**Screening Tool Subscales**					
	Commitment to Research	Personal & Health Benefits	Distrust of Research	Trial Incompatibility	Partner Disclosure	Visit Adherence
**Monitoring Tool Subscales**						
Original Sample						
Vaginal Ring Doubts	-0.04	0.05	0.42	0.67	-0.16	-0.35
Concerns about Side Effects	-0.16	0.02	0.53	0.09*	-0.05	-0.17
Vaginal Ring Removal[a]	-0.04*	0.03*	0.21	0.09	-0.12	-0.25
Vaginal Ring Benefits	0.04	0.3	-0.36*	0.13	0.09	0.37
Current Sample						
Study Pill Challenges	0.08	0.12	0.63	0.51	-0.03	-0.19
Concerns about Side Effects	0.01	0.09	0.38	0.34*	-0.05	-0.16
Positive Pill Adherence[a]	0.41*	0.29*	-0.25	0.04	0.24	0.24
Social Difficulties with Clinical Trial Participation	-0.15	-0.08	0.24	0.21	-0.53	-0.15
Pill Benefits	0.21	0.22	0.05*	0.15	0.09	0.18

*Difference between Pearson Product correlation in original sample and current sample is 0.20 or greater.

[a]Inverse relationship expected between these corresponding subscales because adherence is measured in the opposite direction.

DRAFT

Descriptive statistics for subscale scores are represented for each subscale in the selected solution in Table 7, disaggregated by site and trial participation status.

Table 7. Mean scores for Screening Tool and Monitoring Tool subscales by site and trial experience.

	Site		Trial Experience		Full Sample
	South Africa	Kenya	FTP	NTP	
Screening Tool					
Commitment to Research	5.0	4.9	5.0	5.1	5.0
Personal & Health Benefits	5.2***	4.8***	5.0	5.1	5.0
Distrust of Research	2.7***	2.1***	2.4	2.4	2.4
Trial Incompatibility	3.4***	2.9***	3.1	3.2	3.2
Partner Disclosure	5.0***	4.3***	4.6	4.5	4.6
Visit Adherence	4.9	5.0	4.9	5.0	4.9
Monitoring Tool					
Study Pill Challenges	3.1***	2.5***	2.8	2.7	2.8
Concerns about Side Effects	2.5	2.3	2.3	2.7	2.4
Positive Pill Adherence	4.9	5.0	5.0	4.6	5.0
Social Difficulties with Clinical Trial Participation	2.3***	3.4***	2.7	3.3	2.8
Pill Benefits	5.1*	4.8*	5.1***	3.8***	4.9

*p<0.10 between subgroups; **p<0.05; ***p<0.01

As reflected in table 7, site level differences in mean scale scores were generally larger than any differences by clinical trial experience. South African participants scored significantly higher on most screening tool subscales than did Kenyan participants. These differences may reflect broader cultural factors, or may be due to differences in the socio-demographic variations in our study sample. As might be expected, former trial participants scored significantly higher on

DRAFT

items related to Pill Benefits than women who had not participated in trials previously. There were no other differences in scale scores by trial experience.

Discussion

The validity of psychosocial scales or measurement tools is strengthened to the degree that they perform well in multiple settings and over time [32]. To that end, this research study provided additional evidence that our previously developed screening and monitoring tools performed adequately or better in two new populations of potential and former clinical trial participants in South Africa and Kenya. As oral PrEP clinical trials and public sector rollout of PrEP regimens continue in new settings and with new populations, having reliable tools to screen for potential adherence challenges and track adherence overtime will be vital to ensure that PrEP clinicians and providers can accurately follow product efficacy in these new contexts. These tools add to their ability to do so; this study has further validated the screening tool and expanded the monitoring tool to a wider product scope by adding an oral PrEP-specific version.

Screening Tool

When participants do not use products as instructed within trials, they reduce the potential of the trial to determine safety and efficacy. Although low adherence could indicate a lack of acceptability, the relationship between acceptability and adherence in clinical trial settings is further confounded by the trial design itself. For example, motivations to participate in order to receive reimbursements or access to health care, or the potential to be assigned to a placebo product or the need to adhere to additional trial-related procedures may impact trial participants' behaviors in ways that may not be assessed [33, 34]. Our original screening tool included six

DRAFT

domains that could indicate a potential trial participants' propensity to adhere (9). A theoretical strength of the tool is that the items themselves are product-agnostic. When we evaluated the tool among women who may have participated in oral PrEP studies or had used daily pills for other purposes, the tool performed in a similar way to the original sample. While the "Visit Adherence" subscale was not further evaluated due to its brevity, three of five subscales showed good fit when compared to their previous performance – the "Distrust of Research," "Trial Incompatibility," and "Partner Disclosure" subscales. In contrast, two subscales – "Personal and Health Benefits" and "Commitment to Research" – had lower-than-desirable fit indices, suggesting that participants in this sample may have understood and/or responded to these items differently than in the original study. Further research could be beneficial to refine item content. Nevertheless, the correlations between all of the subscales were consistent with our original assumptions based on the literature [9], providing evidence for their construct validity. An additional limitation is that our tools (both the original vaginal ring and oral PrEP scales) were developed and validated among young adult African women from South Africa and Kenya. Additional studies may be needed to validate their use among adolescent women, men or in other country contexts.

A validated screening tool could be a useful way to flag potential challenges related to product adherence in clinical trial research that stem from trial distrust, partner disclosure or misalignments in personal motivations or life contexts that undermine product use. In a service delivery setting, such domains might be useful to assess when counseling a potential client about the challenges to PrEP adherence – or when determining whether clients can be provided with a greater number of PrEP pill cycles between routine visits. Furthermore, with the approval of

DRAFT

long-acting products, which are less dependent on user adherence, these scales could aid in the shared decision-making and choice of PrEP methods.

Monitoring Tool

This study also provided further support for the structure and content of an oral pill monitoring tool. The monitoring tool items, which were revised to assess perceptions about and experiences using daily oral PrEP, factored into five subscales that showed moderate to good reliability. Four of the five subscales had a similar structure overall to the vaginal ring monitoring tool items from which they were adapted. “Study Pill Challenges” captured similar difficulties in adherence as “Vaginal Ring Doubts.” “Pill Benefits” likewise captured benefits to product use similarly to “Vaginal Ring Benefits.” “Concerns about Side Effects” is nearly product-agnostic and captures a range of concerns applicable to any product. The subscales “Vaginal Ring Removal” and “Positive Pill Adherence” demonstrated the greatest change between products; however, this is expected given the difference between the “active” adherence required of oral PrEP in comparison to the more “passive” adherence of vaginal rings [29, 35, 36]. An additional subscale related to “Social Difficulties of Trial Participation” emerged from the EFA. Given trial participants’ and PrEP users’ reported challenges to adherence due to perceived stigma and issues with disclosure, we believe these items would be useful to monitor over the course of trial participation. In non-trial settings, clients’ assessments of product-related benefits, challenges or adherence-related behaviors, self-administered at the clinic or via an app, could monitor changes in adherence determinants or guide delivery of more targeted counseling or communications messages aimed at supporting adherence prospectively.

DRAFT

Conclusions

Accurately measuring product adherence in HIV-prevention clinical trials and non-trial settings is vitally important in understanding and supporting product efficacy and safety. The effectiveness of a HIV PrEP intervention depends on adequate adherence. Even as new biomarker measures to monitor adherence are developed, measures that explore adherence motivations and challenges in addition to actual product adherence will help researchers develop and bring new products to scale and enhance clinicians' capacity to support product use. Behavioral assessments such as the screening and monitoring tools discussed here are inexpensive, non-invasive, and allow for immediate feedback and the construction of a matrix of different factors of adherence and are likely to continue to be used widely in conjunction with biomarker adherence measures. In this study, we provided further evidence of validation for these measures, demonstrating the screening tool's utility in additional populations and adapting the monitoring tool's utility for different HIV-prevention products. To date, we have not had the opportunity to assess the ability of the screening tool or its domains to predict adherence prospectively. We invite clinical researchers and program implementers to assist in prospectively validating some or all of the domains in the screening and monitoring tools by evaluating their ability to predict adherence patterns as measured by objective biomarkers or other adherence measures in future clinical trials or PrEP-related programs.

Acknowledgements

DRAFT

We would like to acknowledge the willing contributions of the women who participated in this study without whom this study would not have been possible. This project was made possible by the generous assistance from the American people through the U.S. President's Emergency Plan for AIDS Relief (PEPFAR) and the U.S. Agency for International Development (USAID) through a cooperative agreement (AID-OAA-A-14-00011) with CONRAD and Eastern Virginia Medical School. The contents are the responsibility of the authors and do not necessarily reflect the views of PEPFAR, USAID or the United States Government.

DRAFT

References

1. Baeten JM, Donnell D, Ndase P, Mugo NR, Campbell JD, Wangisi J, et al. Antiretroviral prophylaxis for HIV prevention in heterosexual men and women. New England Journal of Medicine. 2012;367(5):399-410.
2. Choopanya K, Martin M, Suntharasamai P, Sangkum U, Mock PA, Leethochawalit M, et al. Antiretroviral prophylaxis for HIV infection in injecting drug users in Bangkok, Thailand (the Bangkok Tenofovir Study): a randomised, double-blind, placebo-controlled phase 3 trial. The Lancet. 2013;381(9883):2083-90.
3. Thigpen MC, Kebaabetswe PM, Paxton LA, Smith DK, Rose CE, Segolodi TM, et al. Antiretroviral preexposure prophylaxis for heterosexual HIV transmission in Botswana. New England Journal of Medicine. 2012;367(5):423-34.
4. Velloza J, Delany-Moretlwe S, Baeten JM. Comprehensive HIV risk reduction interventions for 2020 and beyond: product choices and effective service-delivery platforms for individual needs and population-level impact. Current Opinion in HIV and AIDS. 2019;14(5):423-32.
5. Glidden DV. Advancing Novel PrEP Products–Alternatives to Non-Inferiority. Statistical communications in infectious diseases. 2019;11(1).
6. Montgomery ET, Beksinska M, Mgodi N, Schwartz J, Weinrib R, Browne EN, et al. End-user preference for and choice of four vaginally delivered HIV prevention methods among young women in South Africa and Zimbabwe: the Quatro Clinical Crossover Study. Journal of the International AIDS Society. 2019;22(5):e25283.
7. AVAC. HIV Prevention Research & Development Database. In: AVAC, editor. 2019.
8. Celum CL, Delany-Moretlwe S, Baeten JM, van der Straten A, Hosek S, Bukusi EA, et al. HIV pre-exposure prophylaxis for adolescent girls and young women in Africa: from efficacy trials to delivery. Journal of the International AIDS Society. 2019;22:e25298.
9. Tolley EE, Guthrie KM, Zissette S, Fava JL, Gill K, Louw CE, et al. Optimizing adherence in HIV prevention product trials: Development and psychometric evaluation of simple tools for screening and adherence counseling. PloS one. 2018;13(4):e0195499.
10. Corneli AL, McKenna K, Perry B, Ahmed K, Agot K, Malamatsho F, et al. The science of being a study participant: FEM-PrEP participants' explanations for overreporting adherence to the study pills and for the whereabouts of unused pills. J Acquir Immune Defic Syndr. 2015;68(5):578-84. doi: 10.1097/QAI.0000000000000525. PubMed PMID: 25761233.
11. van der Straten A, Montgomery ET, Musara P, Etima J, Naidoo S, Laborde N, et al. Disclosure of pharmacokinetic drug results to understand nonadherence. AIDS. 2015;29(16):2161-71. doi: 10.1097/QAD.0000000000000801. PubMed PMID: 26544581; PubMed Central PMCID: PMCPMC4638164.
12. Network MT. Daily HIV Prevention Approaches Didn't Work for African Women in the VOICE Study 2012 [March 15, 2013]. Available from: http://www.mtnstopshiv.org/node/4877.
13. Van Damme L, Corneli A, Ahmed K, Agot K, Lombaard J, Kapiga S, et al. Preexposure prophylaxis for HIV infection among African women. N Engl J Med. 2012;367(5):411-22. doi: 10.1056/NEJMoa1202614. PubMed PMID: 22784040; PubMed Central PMCID: PMCPMC3687217.
14. Tolley EE, Harrison PF, Goetghebeur E, Morrow K, Pool R, Taylor D, et al. Adherence and its measurement in phase 2/3 microbicide trials. AIDS Behav. 2010;14(5):1124-36. doi: 10.1007/s10461-009-9635-x. PubMed PMID: 19924525; PubMed Central PMCID: PMCPMC2944966.
15. Agot K, Taylor D, Corneli AL, Wang M, Ambia J, Kashuba AD, et al. Accuracy of Self-Report and Pill-Count Measures of Adherence in the FEM-PrEP Clinical Trial: Implications for Future HIV-Prevention

DRAFT

Trials. AIDS Behav. 2015;19(5):743-51. doi: 10.1007/s10461-014-0859-z. PubMed PMID: 25100053; PubMed Central PMCID: PMCPMC4415940.
16. Husnik MJ, Brown ER, Marzinke M, Livant E, Palanee-Phillips T, Hendrix CW, et al. Implementation of a Novel Adherence Monitoring Strategy in a Phase III, Blinded, Placebo-Controlled, HIV-1 Prevention Clinical Trial. J Acquir Immune Defic Syndr. 2017;76(3):330-7. doi: 10.1097/QAI.0000000000001503. PubMed PMID: 28746164; PubMed Central PMCID: PMCPMC5634926.
17. Haberer JE, Baeten JM, Campbell J, Wangisi J, Katabira E, Ronald A, et al. Adherence to antiretroviral prophylaxis for HIV prevention: a substudy cohort within a clinical trial of serodiscordant couples in East Africa. PLoS Med. 2013;10(9):e1001511. doi: 10.1371/journal.pmed.1001511. PubMed PMID: 24058300; PubMed Central PMCID: PMCPMC3769210.
18. Stalter RM, Moench TR, MacQueen KM, Tolley EE, Owen DH, Consortium for Ring A. Biomarkers and biometric measures of adherence to use of ARV-based vaginal rings. J Int AIDS Soc. 2016;19(1):20746. PubMed PMID: 27144262.
19. Musinguzi N, Muwonge T, Thomas K, Baeten JM, Bangsberg DR, Haberer JE. Does Adherence Change When No One is Looking? Comparing Announced and Unannounced Tenofovir Levels in a PrEP Trial. AIDS Behav. 2016;20(11):2639-43. doi: 10.1007/s10461-016-1292-2. PubMed PMID: 26781865.
20. Brown ER, Hendrix CW, van der Straten A, Kiweewa FM, Mgodi NM, Palanee-Philips T, et al. Greater dapivirine release from the dapivirine vaginal ring is correlated with lower risk of HIV-1 acquisition: a secondary analysis from a randomized, placebo-controlled trial. J Int AIDS Soc. 2020;23(11):e25634. doi: 10.1002/jia2.25634. PubMed PMID: 33206462; PubMed Central PMCID: PMCPMC7673220.
21. Brooks KM, Anderson PL. Pharmacologic-Based Methods of Adherence Assessment in HIV Prevention. Clin Pharmacol Ther. 2018;104(6):1056-9. doi: 10.1002/cpt.1201. PubMed PMID: 30178462; PubMed Central PMCID: PMCPMC6226319.
22. Haberer JE. Current concepts for PrEP adherence in the PrEP revolution: from clinical trials to routine practice. Curr Opin HIV AIDS. 2016;11(1):10-7. doi: 10.1097/COH.0000000000000220. PubMed PMID: 26633638; PubMed Central PMCID: PMCPMC4801217.
23. Lam WY, Fresco P. Medication Adherence Measures: An Overview. Biomed Res Int. 2015;2015:217047. doi: 10.1155/2015/217047. PubMed PMID: 26539470; PubMed Central PMCID: PMCPMC4619779.
24. DeVellis RF. Scale development : theory and applications. Fourth edition. ed. Los Angeles: SAGE; 2017. xvii, 262 pages p.
25. Wu J-R, Chung M, Lennie TA, Hall LA, Moser DK. Testing the psychometric properties of the Medication Adherence Scale in patients with heart failure. Heart & Lung: The Journal of Acute and Critical Care. 2008;37(5):334-43.
26. Johnson MO, Neilands TB, Dilworth SE, Morin SF, Remien RH, Chesney MA. The role of self-efficacy in HIV treatment adherence: validation of the HIV Treatment Adherence Self-Efficacy Scale (HIV-ASES). Journal of behavioral medicine. 2007;30(5):359-70.
27. Morisky DE, Green LW, Levine DM. Concurrent and predictive validity of a self-reported measure of medication adherence. Medical care. 1986:67-74.
28. Bowling A. Just one question: If one question works, why ask several? Journal of Epidemiological Community Health. 2005;59(5):342-5.
29. Tolley E, Friedland B, Gafos M, Amico R, Van Damme L, Woodsong C, et al. Socioeconomic and behavioral factors influencing choice, adherence, and success of microbicide formulations. 2014.
30. Mack N, Evens EM, Tolley EE, Brelsford K, Mackenzie C, Milford C, et al. The importance of choice in the rollout of ARV-based prevention to user groups in Kenya and South Africa: a qualitative study. Journal of the International AIDS Society. 2014;17:19157.

31. Amico KR, Stirratt MJ. Adherence to preexposure prophylaxis: current, emerging, and anticipated bases of evidence. Clinical Infectious Diseases. 2014;59(suppl_1):S55-S60.
32. Brown TA. Confirmatory factor analysis for applied research: Guilford Publications; 2014.
33. Amico KR, Stirratt MJ. Adherence to preexposure prophylaxis: current, emerging, and anticipated bases of evidence. Clin Infect Dis. 2014;59 Suppl 1:S55-60. doi: 10.1093/cid/ciu266. PubMed PMID: 24926036; PubMed Central PMCID: PMCPMC4060253.
34. Woodsong C, MacQueen K, Amico KR, Friedland B, Gafos M, Mansoor L, et al. Microbicide clinical trial adherence: insights for introduction. J Int AIDS Soc. 2013;16:18505. doi: 10.7448/IAS.16.1.18505. PubMed PMID: 23561044; PubMed Central PMCID: PMCPMC3619032.
35. Mastro TD, Sista N, Abdool-Karim Q. ARV-based HIV prevention for women–where we are in 2014. Journal of the International AIDS Society. 2014;17:19154.
36. Amico KR, Wallace M, Bekker L-G, Roux S, Atujuna M, Sebastian E, et al. Experiences with HPTN 067/ADAPT Study-Provided Open-Label PrEP Among Women in Cape Town: Facilitators and Barriers Within a Mutuality Framework. AIDS and Behavior. 2017;21(5):1361-75. doi: 10.1007/s10461-016-1458-y.

DRAFT
