# Supplementary material for: Adaptation and validation of simple tools to screen and monitor for oral PrEP adherence
Source: PLoS One. 2021 May 27;16(5):e0251823. doi: 10.1371/journal.pone.0251823 (PMC8158999; doi:10.1371/journal.pone.0251823)
Supplement: Attachment — Submitted filename: PLoS One_Response to Reviewers.docx [file pone.0251823.s002.docx]

Response to Reviewers

2. Please remove the "DRAFT" that you currently have showing as a watermark in the background of the manuscript pages.

**Response**: This has been done.

 3. We note that you have stated that you will provide repository information for your data at acceptance. Should your manuscript be accepted for publication, we will hold it until you provide the relevant accession numbers or DOIs necessary to access your data. If you wish to make changes to your Data Availability statement, please describe these changes in your cover letter and we will update your Data Availability statement to reflect the information you provide.

**Response**: We have added the link and will activate it once the paper has been published.

Reviewer's Responses to Questions

5. Review Comments to the Author

Please use the space provided to explain your answers to the questions above. You may also include additional comments for the author, including concerns about dual publication, research ethics, or publication ethics. (Please upload your review as an attachment if it exceeds 20,000 characters)

Reviewer #1: The paper touches on an important topic regarding product adherence in clinical trials. The creation of a simple tool that can be used in low resource settings in commendable. The paper however would benefit from a clearer representation of the process undertaken, which could be remedied by a simple schematic to orientate the reader.

**Response**: We clarified the relationship between the “original” study, which was described in the introduction, but had not been directly identified. Information about the original study sample and process can be found in reference 9, which was published in PLoS One in 2018. We believe that the request for a schematic was likely related to confusion about whether the “original” study was separate from, or part of the study described in the manuscript. We hope that our track-changed edits have resolved this lack of clarity.

Additional comments from reviewer 1: Please provide information for how participants recruited.

**Response:** We have now provided that information.

What was meant by “uniformly”?

**Response:** We clarified that, although randomly ordered, the same screening items were administered to all participants (unlike the monitoring items, which were either focused on oral PrEP or other daily oral pills.)

Provide description of the Likert scale.

**Response:** We provided labels for all 6 points on the scale.

Provide information about the “original study” and the “cognitive interviews”.

**Response:** We have added some additional information about the cognitive interviews in the methods. This was a preliminary step to ensure that the screening and monitoring scales were relevant, comprehensible and easy to understand by women in both sites. We do not present additional information on this step because we did not make any major alternations to the items based on this research step.

**Response:**

Reviewer #2: This is an interesting analysis from Tolley and colleagues. I am not an expert on the validation of psychometric scales so I will focus on contextualization and framing of the findings. In spite of the significant challenges that survey adherence scales have experienced in the field of HIV PrEP--I agree they still play an important role. The paper is well written although I think the methods could use some clarification and some additional attention to contextualizing the findings.

Major point 1: Methods: Please provide additional detail in the methods section about the site, design, and context of the original vaginal ring monitoring tool.

**Response**: We are concerned about adding too much additional information about the original study in this manuscript and hope that further clarification linking the last two paragraphs of the introduction section will be sufficient. In addition, we have provided the reference to the original study in this section so that readers can find information about the study design, sample and the initial and finalized items for the two tools.

2. Please also clarify the use of the tool for other product types (re: recruitment of product naive participants). What about many women who have not taken a daily pill prior to PrEP? Would this tool still be administered.

**Response**: The monitoring tool is intended to work with any women who begin using a daily PrEP regimen within a trial setting or in the context of routine care. However, if she were using a different type of product (e.g., vaginal ring or injectable), the monitoring items – because they are product-specific – would need to change. Our original study validated a set of monitoring items that were focused on vaginal ring use. However, we have not developed items related to injections, for example.

3. Intro: You provide a reasonable discussion of the pros and cons of objective vs. self-reported metrics. Is there data that demonstrates that the ring survey correlates with objective adherence? If so I would include it in the introduction or discussion as justification for adapting the scale. Furthermore, ideally this scale would be studied in conjunction with objective adherence metrics as further validation. This will be the major hurdle, given what the field has since learned, in uptake of such a scale.

**Response**: We agree with the reviewer. In fact, the original impetus of the study was to adapt the monitoring tool so that it could be used – and prospectively validated in trials of new PrEP regimens. We are now able to reference in the Conclusion section an upcoming phase 2 trial of Descovy versus Truvada in which we have added an exploratory objective aimed at prospectively validating our tools. We also hope that other clinical researchers will also consider prospectively evaluating the relationship of these tools with objective measures of adherence.

4. Intro: You mention event-drive PrEP but your scale is not set up to address this delivery strategy nor is it currently indicated in this context so I would remove it. White coat adherence is an issue for short-term adherence markers, cumulative metrics are not impacted.

**Response**: Thank you for this comment. We have removed the reference to on-demand PrEP, but also linked the existing references to the hair assay which is currently one of the most promising assays for cumulative adherence.

5. Page 24 line 334. This tool has been presented as a study product monitoring tool, it is not clear to me how it could be used as part of shared decision making between long-acting and other PrEP methods.

**Response**: The screening scales are product-agnostic. However, as the reviewer points out, they are currently oriented towards clinical trial rather than service delivery contexts. Although additional adaptation would be required, some scales – including distrust of product and trial contexts and partner disclosure – are factors influencing adherence to PrEP in routine service contexts. We have modified the text to reflect the need for some adaptation.

6. Page 25 Line 357 and Abstract Conclusions, as no objective adherence metric was included, I don't think you should focus on accuracy during the discussion. I found this confusing when i first read the abstract. I would mention correlation with objective adherence metrics as a next step/limitation

**Response**: We’d like to thank the reviewer for highlighting this point. We have added this as a limitation in the discussion and noted that we have plans to assess the predictive validity of the tool in assessing adherence via objective biomarker. We have also invited other clinical scientists to assist in the tools’ prospective assessment in the conclusion section of the manuscript.

7. Abstract: Need to find space in methods that you adapted the ring tool to measure oral PrEP adherence

**Response**: We feel we have included this information already in the abstract, which states in the Introduction:

In a previous study, we developed tools to screen for general adherence and specifically monitor intravaginal ring adherence within the context of HIV prevention clinical trials. This study aimed to further validate the screening tool and to adapt and provide initial psychometric validation for an oral pill monitoring tool.

Discission P22 line 306: I am not sure that the relationship between adherence and acceptability is "confounded" by the trial design, although the relationship is more complex and has additional inputs. I would try to reformulate this statement to be more clear.

**Response**: Thank you for this comment. We have reworded to clarify that “the relationship … is further *complicated* by the trial *context* itself.”

6. PLOS authors have the option to publish the peer review history of their article ([what does this mean?](https://urldefense.com/v3/__https:/journals.plos.org/plosone/s/editorial-and-peer-review-process*loc-peer-review-history__;Iw!!ELf_LxN3sEQ!ILh1rTwNdzeQwwelu1NwOOa0cFu5fQ6Ys-jM-B4ESu5RNRkAPahyt2_ll2zAV_u1Gg$)). If published, this will include your full peer review and any attached files.


If you choose “no”, your identity will remain anonymous but your review may still be made public.


While revising your submission, please upload your figure files to the Preflight Analysis and Conversion Engine (PACE) digital diagnostic tool, [https://pacev2.apexcovantage.com/](https://urldefense.com/v3/__https:/pacev2.apexcovantage.com/__;!!ELf_LxN3sEQ!ILh1rTwNdzeQwwelu1NwOOa0cFu5fQ6Ys-jM-B4ESu5RNRkAPahyt2_ll2xgQkvZnA$). PACE helps ensure that figures meet PLOS requirements. To use PACE, you must first register as a user. Registration is free. Then, login and navigate to the UPLOAD tab, where you will find detailed instructions on how to use the tool. If you encounter any issues or have any questions when using PACE, please email PLOS at [figures@plos.org](mailto:figures@plos.org). Please note that Supporting Information files do not need this step.
